# Editorial: Perceiving and Acting in the Real World: From Neural Activity to Behavior

**DOI:** 10.3389/fnhum.2016.00179

**Published:** 2016-04-27

**Authors:** Simona Monaco, Gavin Buckingham, Irene Sperandio, J. Doug Crawford

**Affiliations:** ^1^Center for Mind/Brain Sciences, University of TrentoItaly; ^2^School of Life Sciences, Heriot-Watt UniversityEdinburgh, UK; ^3^Sport and Health Sciences, University of ExeterExeter, UK; ^4^School of Psychology, University of East AngliaNorwich, UK; ^5^Centre for Vision Research, York UniversityToronto, ON, Canada

**Keywords:** perception, vision, touch, action, grasping, reaching, saccades

The interaction between perception and action represents one of the pillars of human evolutionary success. Our interactions with the surrounding world involve a variety of behaviors, almost always including movements of the eyes and hands. Such actions rely on neural mechanisms that must process an enormous amount of information in order to generate appropriate motor commands. Yet, compared to the great advancements in the field of perception for cognition, the neural underpinnings of how we control our movements, as well as the interactions between perception and motor control, remain elusive. With this research topic we provide a framework for: (1) the perception of real objects and shapes using visual and haptic information, (2) the reference frames for action and perception, and (3) how perceived target properties are translated into goal-directed actions and object manipulation. The studies in this special issue employ a variety of methodologies that include behavioral kinematics, neuroimaging, transcranial magnetic stimulation, and patient cases. Here we provide a brief summary and commentary on the articles included in this research topic.

## 3D vision for perception and action

Snow et al. (2011) have previously shown that the neural mechanisms involved in the visual processing of 3D real objects differ from those involved in processing 2D images of the same objects. Here, Snow et al. provide behavioral evidence that real-world objects are more memorable than photographs of the same objects. This difference might be related to higher-level attributes that are intrinsic to real but not images of objects, such as affordances for actions, prior associations of a real object with our experience in the world and/or differences in binocular depth cues.

However, binocular vision is not always necessary for movement control. When we perform actions, such as grasping an object or hitting a ball, we normally have binocular vision of the goal and the surrounding scene. Despite the potential advantages of binocular vision, monocular viewing provides sufficient information to engage in online control to correct initial errors in movement planning (Brenner et al.; Gnanaseelan et al.).

## Reference frames for visual localization and aiming movements

The accurate localization of a target in the surrounding environment is essential for skilled aiming movements. There are two general types of reference frames that can be used in order to localize objects in the extra-personal space: allocentric and egocentric. While allocentric frames of reference allow us to encode the location of a target relative to contextual cues in the outside world, egocentric frames of reference allow us to encode the location of a target relative to one's self. Reliance on these frames of reference has been shown to depend on the demands of the spatial task (Taghizadeh and Gail) and on the nature of the task itself (Fiehler et al.). Indeed, while perceptual tasks—generally associated with the ventral visual stream and object-centered coding—are affected by visual illusions, tasks that involve an action—generally associated with the dorsal visual stream and egocentric coding—are not (Dassonville et al.). However, Filimon et al. suggests that all spatial comparisons, even those between different allocentric cues, are ultimately processed in the brain with respect to the self, and therefore involve egocentric frames of reference. If so, this would require a re-consideration of schemes that separate ventral vs. dorsal stream vision on the basis of allocentric vs. egocentric processing.

## Aiming movements of the eyes and hand

Behavioral dissociations between the control of reach direction and amplitude have been recognized for decades (Soechting and Flanders, 1992). However, a corresponding dissociation between the neural mechanisms for reach direction and depth—past early visual cortex—remains controversial. Here, Davare et al. provide evidence for a double-dissociation between direction and amplitude coding for reaching movements within the fronto-parietal reaching circuit in humans. While aIPS is involved in processing the direction of movements, dPM processes the amplitude of movements.

Saccades are often tested in paradigms where their past history is disregarded. However, Jones et al. show that the direction of prior saccades affects the direction of current saccades. Finally, oculomotor physiologists are familiar with studies where visual information is remapped in eye-centered coordinates to compensate for saccades. However, when the saccade target is located on the body, a more complex series of egocentric reference frame transformations is required to account for the position and motion of that body part. Buchholz et al. demonstrate that tactile remapping for saccades induces alpha and beta oscillations that prepare the brain for the upcoming eye-movement based on eye- and body-centered frames of reference.

## Grasp control and kinematics

Grasping movements have been extensively studied over the past two decades, and human neuroimaging as well as cell recording in macaques have allowed unveiling the neural mechanisms underlying actions (for a review see Turella and Lingnau). The two main components of grasping movements consist of reaching the target location and pre-shaping the fingers according to the shape, size and orientation of the target object. Begliomini et al. show that although the involvement of dorsal visual stream areas in reaching and grasping depends on the temporal progression of the movement, similar areas are sensitive to both types of movements, suggesting that the neural underpinnings of reaching and grasping may overlap in both in spatial and temporal terms.

The intimate relationship between the visual system and grasp control is further illustrated by its dependence on field of view. For instance, the motor control of an action is facilitated when the object to be acted upon is in the same visual hemifield of our hand. In particular, right-handed participants scale their grip aperture more accurately to objects placed on their right visual field when grasping with the right hand. Similarly, participants scale their grip aperture more accurately to objects placed on the left visual hemifield when grasping with the left hand (Le and Niemeier).

Finally, grasp is also influenced by the effector and intended use of the object. In particular, Quinlan et al. report that the kinematics of biting a piece of food, which in essence consists of grasping the piece of food with the mouth, differ from those of grasping with the hand. In particular, participants oversize the mouth to a lesser extent when biting than the hand when grasping the same-sized piece of food. The use of a tool, such as a fork, also affects the movement kinematics by slowing down hand movements while leaving the grip component unchanged.

## Haptic contribution to perception and action

Although vision is the sense that we most use in order to perceive objects in our environment, haptic feedback is also crucial for manual exploration and grasping movements. For example, Whitwell et al. show that the removal of haptic feedback at the end of a grasping movement causes higher reliance on vision and cognitive supervision, resulting in grasps that appear to be more like pantomimed movements.

When vision is degraded or unavailable, we often use touch in order to explore and recognize objects in our environment. The network of areas involved in haptic exploration includes much of the cerebral cortex, ranging from occipital areas to temporal and fronto-parietal cortices. In particular, Marangon et al. find that haptic exploration engages ventral visual stream areas (including the lateral occipital area, LO) known to be involved in visual recognition of objects. Marangon et al. further show that LO is involved in exploring and grasping shapes regardless of their complexity, whereas the anterior intraparietal sulcus (aIPS), a dorsal stream area, is more involved in performing grasping movements toward complex vs. simple shapes that have been haptically explored. It has been previously suggested that grasps of increased complexity toward visually explored objects, like tools, require the recruitment of ventral stream areas (van Polanen and Davare, 2015). Therefore, it is possible that the extent to which ventral and dorsal stream areas are recruited during actions toward complex shapes depends on the sensory modality used to explore the shape, with higher involvement of ventral stream areas when the object has been seen and higher involvement of dorsal stream areas when the object has been haptically explored.

## Multi-sensory calibration and motor learning

Since vision is such a dominant sense, it affects the motor and proprioceptive systems even when only limited visual information is available. Indeed, Barkley et al. provide evidence that brief exposure to altered vision of one's arm position in the environment induces motor adaptation and proprioceptive recalibration, resulting in the matching of proprioception with the misaligned visual feedback. This study highlights the powerful influence of vision on both multi-sensory calibration and the dynamics of motor learning.

Successful interactions with the external world includes the ability to accurately predict the forces necessary to lift and manipulate objects in our environment. However, such actions are often subject to perturbations that can be caused by either internal or external factors. For example, the act of walking can be rendered more effortful by an internal factor, such as tiredness, or an external factor, such as walking against the direction of the wind. To make sure that learning happens in the appropriate context, it is important that perturbations are correctly attributed to the right source. In their paper, Fercho et al. contribute to this topic by showing that the rate of perturbation that is experienced by participants while lifting an object plays a critical role in how the motor system solves the credit assignment problem and consequently, motor adaptation effects for subsequent lifting actions.

## Sensorimotor coordination

Bimanual coordination is required for many daily activities, ranging from simple tasks such as peeling an orange, to more complex learned tasks like playing the piano. In these examples, the hands concurrently perform different movements, each with differing temporal and spatial demands. Garbarini et al. explored the neural correlates of congruent and non-congruent bimanual coordination in patients with motor neglect. Congruent movements consisted of performing the same drawings (lines or circles) with both hands, while non-congruent movements required participants to perform different movements with the two hands (line drawings with the right hand and circle drawings with the left hand). The lack of interference between the motor programs of two hands during non-congruent bimanual movements (an effect observed in individuals with motor neglect) is associated with decreased activation in pre-supplementary motor area (pre-SMA) as compared to congruent bimanual movements. Control participants (with and without brain damage) showed the opposite pattern, with higher activation in pre-SMA for non-congruent vs. congruent bimanual coordination. These results suggest that the lack of inhibition exerted by pre-SMA might be at the basis of the behavioral impairment during non-congruent bimanual coordination in patients with motor neglect. This might be related to the role of pre-SMA in in processing the flow of information between the two hemispheres in order to control for interference between the motor programs of the two hands during bimanual coordination.

Parkinson's disease (PD) is associated with basal ganglia dysfunction and a number of symptoms, including deficits in muscle co-activation (i.e., synergies). In particular, van der Stouwe et al. demonstrate that decreased performance in composite arm movements in PD patients is associated with decreased activity in the striatum and in the fronto-parietal network. This highlights the need to understand interactions between sub-cortical and cortical disease processes such as PD.

Finally, arm movements are not controlled in isolation from the rest of the body. Indeed, the trunk provides the postural basis for reach. Effects of controlling the upper and lower regions of the trunk during reaching provides insight into the mechanisms by which trunk control impacts reaching in infants. Trunk control is acquired in a segmental sequence across development of upright sitting and it is tightly correlated with reaching performance (Rachwani et al.)

## Action observation

Actions allow us to interact not only with objects in our environment but also with other people. During social interactions, we observe other people's movements which in turn activate our own motor system through a process known as “motor resonance.” Motor resonance is differently affected by hand dominance (Sartori et al.), suggesting that the motor system is fine-tuned not only to our own actions but also to other people's actions. In addition, Balser et al. provide evidence that, during action observation, brain activity in the fronto-parietal network correlates with performance in sport experts when anticipating the effects of actions performed by others in their preferred discipline.

## Conclusions

This research topic outlines a number of recent advances in our understanding of the neural mechanisms and the associated behavior for perception and action. In this review, we have emphasized the intimate relationship between perceptual motor systems, not only in the obvious sense that sensation can be used to guide action, but in the many ways that perception and action interact, up to and including the perception of actions in others. Further, the many examples cited above illustrate the clear link between this topic and applications for real world behavior; not only for clinical populations and elite athletes, but in nearly every aspect of our waking lives. For this, we are grateful to all of the authors and reviewers that contributed to the composition of this special topic issue.

## Author contributions

SM, JC contributed to the design of the work and drafted the editorial. GB, IS revised the draft for important intellectual content and contributed with the interpretation of the work. SM, GB, IS, JC approved the final version to be published and agreed to be accountable for all aspects of the editorial in ensuring that questions related to the accuracy or integrity of any part of the work are appropriately investigated and resolved.

## Funding

Canada Research Chairs, Canadian Institutes of Health Research, Natural Sciences and Engineering Research Council of Canada to JC.

### Conflict of interest statement

The authors declare that the research was conducted in the absence of any commercial or financial relationships that could be construed as a potential conflict of interest.
